# The intelligibility of *r* or *r*^2^ as an effect size statistic: dichotomous variables

**DOI:** 10.3389/fpsyg.2015.00294

**Published:** 2015-03-17

**Authors:** David Trafimow

**Affiliations:** Department of Psychology, New Mexico State UniversityLas Cruces, NM, USA

**Keywords:** correlation coefficient, coefficient of determination, adjusted proportion of successes, adjusted success rate, proportion of successes

There have been differences in the use of the correlation coefficient (*r*) or the coefficient of determination (*r*^2^) for indexing the effect size (see Rosenthal and DiMatteo, 2001; Borenstein, 2009; Elis, 2010, for reviews). I intend to investigate this issue by considering it from the point of view of matching the findings with the implied prediction. In essence my argument follows from a simplification of the correlation coefficient to the case where both variables are dichotomous and where there are equal frequencies of each possible response on both variables. Based on this simplified case, the question is whether the correlation coefficient or the coefficient of determination most closely resembles the actual proportion of agreements (successes) between the two variables after controlling for chance.

To flesh out the idea, suppose that there are two variables and each of these is dichotomous and scored 0 or 1. From the point of view of a researcher who believes that the relation between the two variables is important, each case of matching scores (0 on both variables or 1 on both variables) is a success whereas each case of mismatching (0 on one variable and 1 on the other, or the reverse) constitutes a failure. The straightforward way to index the ability of the two variables to produce successes (agreements with respect to zeroes and ones) would be to use the proportion of obtained successes. However, because a 50% success rate would be expected due to chance, this proportion likely would be misleading.

I suggest controlling for chance by computing an adjusted proportion of successes or adjusted success rate (*S*_*A*_) using Equation (1) below, where *s* refers to the proportion of successes and *C* refers to the proportion of successes that would be expected based on chance alone.

(1) $$S_{A}=\frac{s-C}{1-C}$$

In correlation terms, given the simplification mentioned previously, the usual phi correlation coefficient reduces to the equation made famous by Rosenthal and Rubin (1982) rendered below as Equation (2), where *r* denotes the correlation between the two variables.

(2) $$s=0.5+\frac{r}{2}$$

Substituting Equation (2) into Equation (1) renders Equation (3).

(3) $$S_{A}=\frac{\left(0.5+\frac{r}{2}\right)-C}{1-C}$$

Remembering that when there are two variables, we expect a 50% success rate by chance, 0.5 can be substituted for *C* rendering Equation (4).

(4) $$S_{A}=\frac{\left(0.5+\frac{r}{2}\right)-0.5}{1-0.5}$$

In turn, Equation (4) simplifies to Equation (5).

(5) $$S_{A}=r$$

Put into words, in the dichotomous case when there are equal numbers of zeroes and ones for both variables, the success rate adjusted for chance equals the correlation coefficient!

In summary, then, my argument is simple. Because the proportion of successes, controlling for chance, is a straightforward and easy way to understand an effect size, this should be the preferred effect size statistic. Happily, the correlation coefficient equals this under the simplified conditions that I set up. Therefore, in terms of straightforward intelligibility, the correlation coefficient is superior to the coefficient of determination as an effect size index.

Although my main point has been made, there are additional issues worth mentioning. First, there are additional reasons to favor *r* over *r*^2^. One such reason is that the former is directional whereas the latter is not. Another reason is that *r* has a straightforward interpretation in terms of standardized slope (the implications that a change in one variable has for a change in the other). Thus, Equation 5 is not the only reason to favor *r* over *r*^2^.

A second issue is that it is possible for *r* to be a problematic measure of effect size even though it is superior to *r*^2^. Baguley (2009) contrasted standardized vs. unstandardized effect size measures. Both *r* and *r*^2^ are standardized effect size measures and the reliabilities of the measures of the variables have a strong influence on standardized effect size measures. As reliabilities decrease standard deviations increase, and so effect size measures that are standardized via standard deviations (in the denominator) decrease. For those researchers who wish to have their effect size measures uninfluenced by reliability issues, they either can use the famous correction formula from classical test theory or use an effect size measure that is not standardized. Each of these involves considerations that go beyond the present scope.

The final issue I will consider pertains to the use of the present logic when one is considering correlation coefficients that are not based on dichotomous data with equal frequencies. To address this issue, it is important to remember that Equation 2 played an important role in getting to Equation 5 and that there has been much discussion about it in the literature. Rosenthal and Rubin (1982) and Rosenthal et al. (2000) argued that there usually is a tolerable amount of distortion when Equation (2) is applied outside the restricted domain involving dichotomous data with equal frequencies whereas Hsu (2004) suggested that there is an important amount of bias when the frequencies (or variances) are too unequal. A possible compromise conclusion is that generalization of Equation (2) outside the present case is justifiable when frequencies or variances are reasonably similar but not when they are extremely dissimilar.

## Conflict of interest statement

The authors declare that the research was conducted in the absence of any commercial or financial relationships that could be construed as a potential conflict of interest.
